# Osmolyte regulation by TonEBP/NFAT5 during anoxia-recovery and dehydration–rehydration stresses in the freeze-tolerant wood frog (*Rana sylvatica*)

**DOI:** 10.7717/peerj.2797

**Published:** 2017-01-19

**Authors:** Rasha Al-attar, Yichi Zhang, Kenneth B. Storey

**Affiliations:** Institute of Biochemistry, Departments of Biology and Chemistry, Carleton University, Ottawa, ON, Canada

**Keywords:** Nuclear factor of activated T cells, *Rana sylvatica*, Dehydration, Anoxia, Immunoblotting, Osmotic stress

## Abstract

**Background:**

The wood frog, *Rana sylvatica*, tolerates freezing as a means of winter survival. Freezing is considered to be an ischemic/anoxic event in which oxygen delivery is significantly impaired. In addition, cellular dehydration occurs during freezing because water is lost to extracellular compartments in order to promote freezing. In order to prevent severe cell shrinkage and cell death, it is important for the wood frog to have adaptive mechanisms for osmoregulation. One important mechanism of cellular osmoregulation occurs through the cellular uptake/production of organic osmolytes like sorbitol, betaine, and myo-inositol. Betaine and myo-inositol are transported by the proteins BGT-1 and SMIT, respectively. Sorbitol on the other hand, is synthesized inside the cell by the enzyme aldose reductase. These three proteins are regulated at the transcriptional level by the transcription factor, NFAT5/TonEBP. Therefore, the objective of this study was to elucidate the role of NFAT5/TonEBP in regulating BGT-1, SMIT, and aldose reductase, during dehydration and anoxia in the wood frog muscle, liver, and kidney tissues.

**Methods:**

Wood frogs were subjected to 24 h anoxia-4 h recovery and 40% dehydration-full rehydration experiments. Protein levels of NFAT5, BGT-1, SMIT, and aldose reductase were studied using immunoblotting in muscle, liver, and kidney tissues.

**Results:**

Immunoblotting results demonstrated downregulations in NFAT5 protein levels in both liver and kidney tissues during anoxia (decreases by 41% and 44% relative to control for liver and kidney, respectively). Aldose reductase protein levels also decreased in both muscle and kidney tissues during anoxia (by 37% and 30% for muscle and kidney, respectively). On the other hand, BGT-1 levels increased during anoxia in muscle (0.9-fold compared to control) and kidney (1.1-fold). Under 40% dehydration, NFAT5 levels decreased in liver by 53%. Aldose reductase levels also decreased by 42% in dehydrated muscle, and by 35% in dehydrated liver. In contrast, BGT-1 levels increased by 1.4-fold in dehydrated liver. SMIT levels also increased in both dehydrated muscle and liver (both by 0.8-fold).

**Discussion:**

Overall, we observed that osmoregulation through an NFAT5-mediated pathway is both tissue- and stress-specific. In both anoxia and dehydration, there appears to be a general reduction in NFAT5 levels resulting in decreased aldose reductase levels, however BGT-1 and SMIT levels still increase in certain tissues. Therefore, the regulation of osmoregulatory genes during dehydration and anoxia occurs beyond the transcriptional level, and it possibly involves RNA processing as well. These novel findings on the osmoregulatory mechanisms utilized by the wood frog advances our knowledge of osmoregulation during anoxia and dehydration. In addition, these findings highlight the importance of using this model to study molecular adaptations during stress.

## Introduction

Extreme environmental conditions such as freezing temperatures, water loss, food shortage, as well as prolonged exposure to minimal or no oxygen, can impose significant distress to organisms in the wild. Many amphibians live in environments where they are confronted by these stresses, and in order to survive, they have developed a range of biochemical and physiological adaptations that aid in long-term survival. Wood frogs (*Rana sylvatica*) tolerate freezing as a mechanism to survive winters. In fact, they are able to tolerate freezing of 65–70% of total body water, which accumulates in extracellular and extraorgan ice masses (Storey & Storey, 1996; Storey & Storey, 2013). Wood frogs endure the accumulation of ice crystals throughout their bodies, which interfere with vital physiological functions such as neuronal activity, muscle movement, breathing, circulation, digestion, and waste filtration (Layne & First, 1991; Kling, Costanzo & Lee, 1994; Storey & Storey, 2004a). In addition to a need to manage ice formation, frozen frogs need to cope with reduced or no oxygen delivery to their tissues (since heart rate and blood flow stop) and severe dehydration when water is lost from cells to promote freezing of the extracellular compartments. Wood frogs have well-developed anoxia resistance (at least 48 h exposure to N_2_ gas at 5 °C), and dehydration resistance (ability to endure the loss of approximately 60% of total body water) (Churchill & Storey, 1994; Holden & Storey, 1997; Storey & Storey, 2004b). Therefore, understanding how these animals endure these stresses naturally has important biological and biomedical implications as these stresses are tolerated poorly by mammals, including humans.

Wood frogs have developed various adaptations that allow it to effectively combat anoxia/ischemia and extreme cellular dehydration. One important mechanism used by wood frogs is the accumulation of glucose as a cryoprotectant and osmolyte. For example, glucose levels can increase approximately 20-fold in skeletal muscle in frozen frogs compared to unfrozen frogs in order to prevent severe cell shrinkage and cell death (Storey & Storey, 1984). In addition, urea concentrations increase as this osmolyte is known to accumulate under dehydration stress in amphibians and it helps to slow the loss of body fluids that would otherwise lead to injury/death caused by reduced blood volume and viscosity (Costanzo & Lee, 2005; Costanzo & Lee, 2008). Adaptations to stresses including dehydration and anoxia involve profound biochemical and molecular changes at the transcriptional, post-transcriptional, translational, and post-translational levels (Roufayel, Biggar & Storey, 2011; Sullivan, Biggar & Storey, 2014; Gerber, Wijenayake & Storey, 2016). Although much has recently been discovered about how frogs adapt to stress on a molecular level, there is much that remains undiscovered, especially in the wood frog.

As discussed previously, freezing, dehydration, and anoxia can cause water loss from cells and into extracellular compartments. Therefore, the cells are under hypertonic stress, and in order to avoid significant shriveling and shrinking that leads to cell death, cells elicit a genetic program of osmoregulation. This response gradually replaced electrolytes like Na^+^, Cl^−^, and K^+^ by small, uncharged organic osmolytes like sorbitol, betaine, and myo-inositol (Yancey et al., 1982; Garcia-Perez & Burg, 1991). These organic osmolytes play an integral role in osmoregulation due to their ability to accumulate without disturbing cellular structure and function. In fact, when kidney-derived Madin-Darby canine kidney (MDCK) cells were cultured in hypertonic solution, the concentration of betaine in the cell increased to 1,000 times the medium concentration (Yamauchi et al., 1992). Therefore, organic osmolytes accumulate as a response to cell shrinkage, and they are released following swelling (Burg & Ferraris, 2008; Lambert & Hansen, 2011). Different enzymes and transporters are responsible for the accumulation of these osmolytes in the cell. Sorbitol is synthesized by aldose reductase, whereas betaine and myo-inositol are transported into the cell by betaine transporter (BGT-1) and sodium/myo-inositol cotransporter (SMIT), respectively (Burg, Kwon & Kültz, 1997; Gallazzini et al., 2006).

Gene expression of these of the osmoregulatory genes are increased by cell shrinkage in a hypertonic environment. Upregulation of gene transcription is carried out by multiple enhancers known as the osmotic-reponse element (ORE) or tonicity-response enhancer (TonE) that are located in the regulatory domains of these genes (Takenaka et al., 1994; Ferraris et al., 1996; Ko et al., 1997). The original transcription factor for ORE/TonE was identified as TonE-binding protein (TonEBP)/ORE-binding protein (OREBP), which shows sequence homology to a family of transcription factors called the Nuclear Factor of Activated T cells (NFAT), and therefore it was named NFAT5 (Lopez-Rodríguez et al., 1999; Miyakawa et al., 1999; Ko et al., 2000). NFAT5 has been discovered to be ubiquitously expressed throughout the body and has been shown to be sensitive to hypertonicity in all cell types (Lopez-Rodríguez et al., 1999; Trama et al., 2000; Franchi-Gazzola et al., 2001; Zhang et al., 2003; Maallem et al., 2006; Tsai et al., 2006; Küper, Beck & Neuhofer, 2015). However, NFAT5-dependent expression of aldose reductase, BGT1, and SMIT is especially important for the survival of cells in the kidney, as these cells are exposed to high and varied levels of sodium and urea (Miyakawa et al., 1998; Na et al., 2003; López-Rodríguez et al., 2004). In addition, NFAT5 is important in dehydration natriuresis by regulating the expression of serum- and glucorticoid-inducible kinase (Sgk1) (Chen et al., 2009). NFAT5-deficient mice develop renal atrophy and most passed away at around 10 days after birth (López-Rodríguez et al., 2004). Therefore, NFAT5-mediated expression of aldose reductase, BGT-1, and SMIT are important for preserving cellular structure and function in kidney cells, if not all cells in the body.

Given the unique ability of *R. sylvatica* to cope with anoxia/ischemia and severe dehydration, there is a need to study the molecular mechanisms underlying osmoregulation during each of these stresses in this animal. We hypothesize that there will be a similar, coordinated response in regulating aldose reductase, BGT-1, and SMIT expression through an NFAT5-mediated mechanism in both stresses as an adaptive mechanism to combat anoxia and dehydration. However, we are also evaluating the organ/tissue-specific responses in the kidney, liver, and skeletal muscle of wood frogs, and we predict that there will be differences in the regulation of the NFAT5-mediated osmoregulatory response as other studies have identified differential expression of these proteins between tissues (Zhang et al., 2003; Maallem et al., 2006; Tsai et al., 2006). To test these hypotheses, the present study characterized the total protein levels of NFAT5, aldose reductase, BGT-1, and SMIT in *R. sylvatica* skeletal muscle, liver, and kidney tissues before and after experimental induction of anoxia-recovery from anoxia as well as severe dehydration-full rehydration conditions.

## Materials and Methods

### Animals

Active and mature male wood frogs (*R. sylvatica*, 210 frogs) were sampled from meltwater ephemeral ponds in the lightly wooded areas near Oxford Mills, Ontario, Canada during the spring and were transported, on ice, to an animal care facility at Carleton University. All the frogs were washed in a tetracycline bath and then held at 5 °C in containers with sphagnum moss for two weeks prior to experimentation. 90 control (normoxic, hydrated, euthermic) frogs were sampled directly from this condition. All animal care, experimentation and euthanasia procedures are approved by the Carleton University Animal Care Committee (animal protocol no. 13683) in accordance to the guidelines set forth by the Canadian Council on Animal Care.

### Anoxic treatment

For anoxia experiments, sealed plastic containers (700 mL) with damp paper towel (wetted with distilled water that was previously bubbled with 100% N_2_ gas for approximately 30  min) were chilled with ice. The lid to the plastic container has two ports: one to allow nitrogen gas in and one to vent the air. The jar was flushed with N_2_ gas for 15–20 min. 60 adult frogs that were acclimated to 5 °C were placed in these containers (6–8 frogs/container) and the lid was tightened and sealed with parafilm. N_2_ gas was then passed through the containers for approximately 30 min and they were placed in 5 °C incubators for 24 h. After the 24 h exposure, half of the containers were placed on ice while still being sealed. N_2_ gas was then reintroduced to the plastic containers and the frogs were quickly sampled with minimal oxygen exposure. This group is the 24 h anoxia group. The other half of the 24 h anoxic frogs were transferred to a new container, where they were exposed to air and allowed to recover from anoxia for 4 h at 5 °C. The 4 h recovery group was sampled immediately after 4 h at 5 °C. All animals were euthanized by pithing, and the liver, kidney, and hindlimb thigh muscle were dissected and immediately flash frozen in liquid nitrogen. Tissues were kept frozen at −80 °C until use.

### Dehydration treatment

Dehydration experiments were performed according to the protocol as previously described by Churchill and Storey (Churchill & Storey, 1994). After acclimation at 5 °C, 60 frogs were individually weighed and ranked from heaviest to lightest and were placed in plastic containers that were held on ice until there were 8–10 frogs of different masses in each container. These containers contained silica gel desiccant on the bottom that was separated from the frogs by a 1 cm thick sponge. The containers were placed in an incubator set at 5 °C where frogs could lose water through evaporation. At varying intervals, the frogs were quickly removed and weighed. The amount of body water lost and regained was calculated using the equation (*M*_*i*_ − *M*_*d*_)∕(*M*_*i*_ × %H_2_O) where *M*_*i*_ is the initial mass of the animal, *M*_*d*_ isthe mass at each weighing, and %H_2_O is the percentage of total body mass that is water (for control wood frogs, %H_2_O is 80.8 ± 1.2%) (Churchill & Storey, 1994). The mean rate of body water loss under these experimental conditions was approximately 0.5% total body water per hour. Experimental dehydration was continued until the frogs reached a 40% loss of total body water and then half of the frogs were sampled. The rest of the dehydrated frogs were allowed to rehydrate until they regained the total water mass that they lost. All animals were euthanized and sampled the same way as the frogs exposed to anoxia.

### Total protein extract preparation

Total protein extracts were prepared as previously described (Zhang & Storey, 2015). Samples of frozen skeletal muscle, liver, and kidney (*n* = 4 individual animals) weighing approximately 500 mg were powdered for the control, 24 h anoxia, 4 h recovery, 40% dehydration, and full rehydration conditions under liquid nitrogen. Samples were homogenized in 1:2 w/v of homogenization buffer (20 mM Hepes, 200 mM NaCl, 0.1 mM EDTA, 10 mM NaF, 1 mM Na_3_VO_4_, 10 mM *β*-glycerophosphate at a pH of 7.5) with 1 mM phenylmethylsulfonyl fluoride (Bioshop) and 1 µL of Sigma protease inhibitor (cat. No. P1Coo1.1; Sigma, Burlington, ON, Canada) using a Polytron PT10 homogenizer for ∼15–20 s. All of the homogenates were then centrifuged at 10,000 rpm at 4 °C for 10 min and the resulting supernatants containing the soluble proteins were collected. The concentrations of the supernatants were measured using a BioRad reagent (Cat #500-0006; BioRad Laboratories, Hercules, CA) at 595 nm on a MR5000 microplate reader. Afterwards, all of the liver, as well as the control skeletal muscle and kidney samples were normalized to 10  µg/µL using homogenization buffer. The dehydration–rehydration and anoxia-recovery muscle samples were normalized to 5  µg/µL. The dehydration–rehydration kidney samples were normalized to 8  µg/µL, and the anoxia-recovery kidney samples were normalized to 2  µg/µL. Then aliquots were combined 1:1 v:v with 2x SDS loading buffer (100 mM Tris-base, pH 6.8, 4% w:v SDS, 20% v:v glycerol, 0.2% w:v bromophenol blue, 10% v:v 2-mercaptoethanol) and the samples were then boiled. The final protein samples at their respective concentrations were stored at −20 °C until use.

### Western blotting

Equal amounts of protein from each sample were loaded onto 6% (NFAT5), 8% (SMIT), 10% (BGT-1), or 15% (aldose reductase) polyacrylamide gels and were run at 180 V for 60–90 min. Proteins were then transferred to PVDF membranes by electroblotting at 160 mA for 45 min (aldose reductase), 90 min (BGT-1), 120 min (SMIT), or 180 min (NFAT5) using a transfer buffer containing 25 mM Tris (pH 8.5), 192 mM glycine and 10% v:v methanol at room temperature. Membranes were then blocked for 30 min with 5% w:v milk in 1x TBST (20 mM Tris base, pH 7.6, 140 mM NaCl, 0.05% v:v Tween-20, 90% v:v ddH_2_O). After washing for 3 × 5 min again with 1x TBST, membranes were probed with specific primary antibodies at 4 °C overnight. Antibodies specific for NFAT5 (sc-13035), BGT-1 (sc-241911), SMIT (sc-48743), and aldose reductase (sc-33219) were purchased and used at a 1:1000 v:v dilution in 1x TBST. After probing with primary antibody, membranes were washed for 3 × 5 min with 1x TBST and then incubated with either HRP-linked anti-rabbit or HRP-linked anti-goat IgG secondary antibody (Bioshop: 1:6000 v:v dilution) for 30 min at room temperature. After a second set of three washes, bands were visualized by enhanced chemiluminescence (H_2_O_2_ and Luminol). Then, blots were restained using Coomassie blue (0.25% w/v Coomassie brilliant blue, 7.5% v/v acetic acid, 50% methanol) to visualize total protein levels.

### Quantification and statistics

Band densities on chemiluminescent immunoblots were visualized using a Chemi-Genius BioImaging system (Syngene, Frederick, MD, USA) and quantified using the Gene Tools software. Immunoblot band density in each lane was standardized against the summed intensity of a group of Coomassie-stained protein bands in the same lane; this group of bands was chosen because they were not located close to the protein band of interest but were prominent and constant across all samples. This method of standardizing against a total protein loading control has been suggested to be more accurate in comparison with standardizing against housekeeping proteins such as tubulin (Eaton et al., 2013). Western blot band densities were normalized at each condition relative to control. Immunoblotting data are expressed as means ± SEM, *n* = 4 independent samples from different animals. Statistical testing used the one-way ANOVA and the Tukey post-hoc functions from the GraphPad Prism software (San Diego, CA, USA).

**Figure 1:**
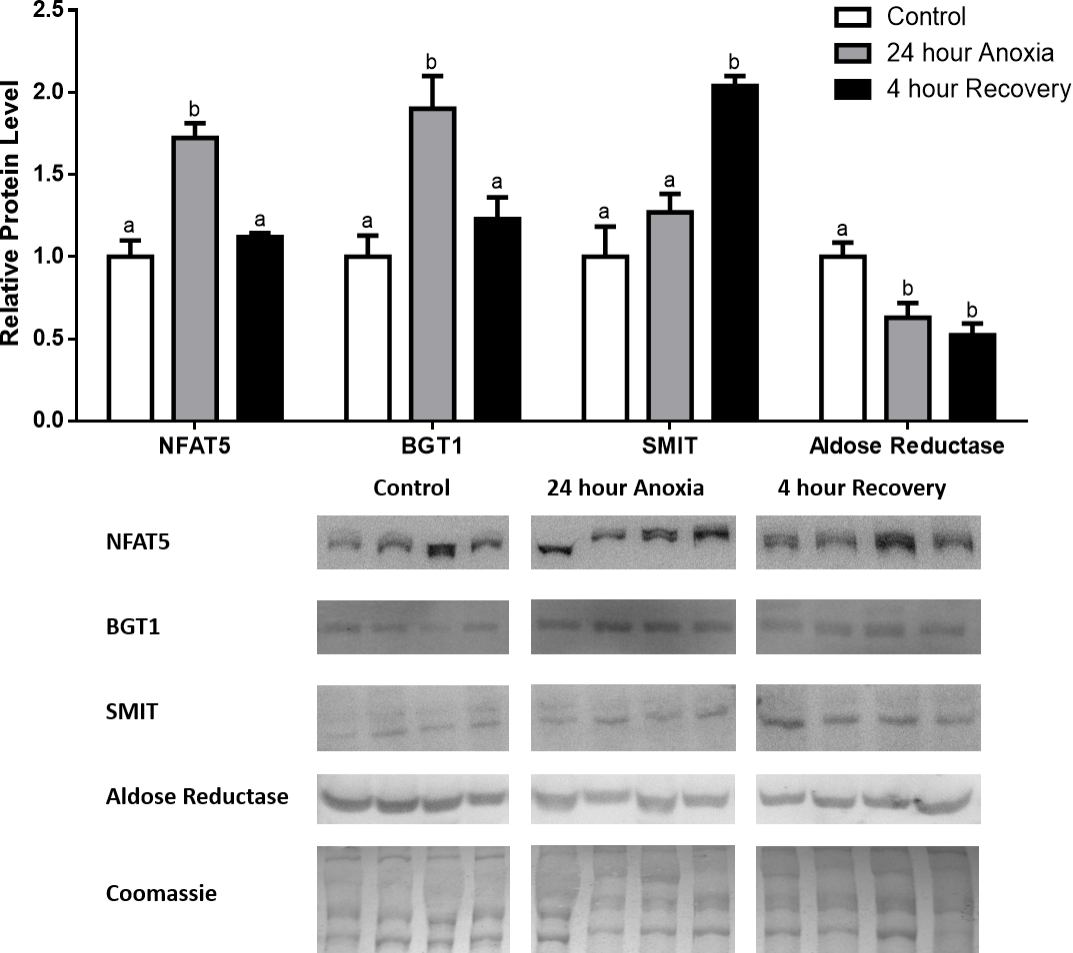
Changes in NFAT5, BGT-1, SMIT, and aldose reductase total protein levels in muscle during anoxia-recovery stress in *R. sylvatica*. Muscle NFAT5, BGT-1, SMIT, and aldose reductase total protein levels were visualized at three sampling points: control, 24 h anoxia, and 4 h recovery. See ‘Materials and Methods’ for more extensive definitions of the sampling points. Westerns blots and Coomassie total protein loading controls representative of the results are shown for the three sampling points. Also shown are histograms with mean standardized band densities (±S.E.M., *n* = 4 independent protein isolations from different animals). Data was analyzed using a one-way analysis of variance with a post hoc Tukey’s test (*p* < 0.05); for each parameter measured, values that are not statistically different from each other share the same letter notation. NFAT5, Nuclear Factor of Activated T Cells 5; BGT-1, betaine transporter 1; SMIT, sodium/myo-inositol cotransporter.

**Figure 2:**
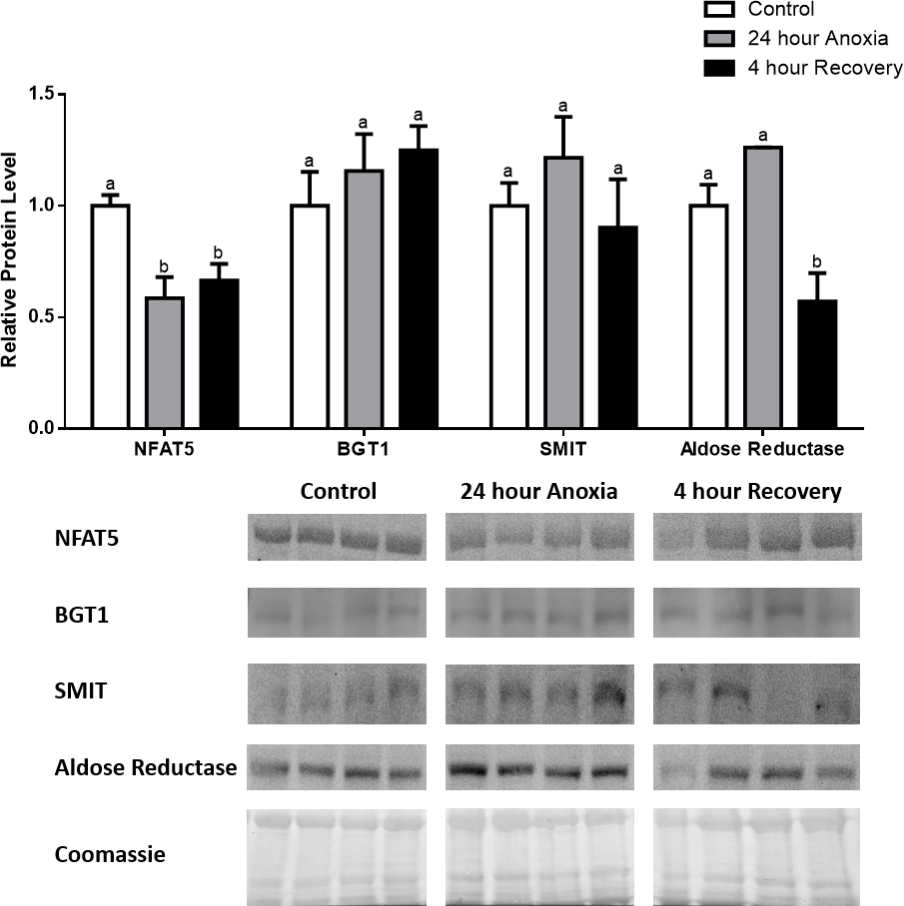
Changes in NFAT5, BGT-1, SMIT, and aldose reductase total protein levels in liver during anoxia-recovery stress in *R. sylvatica*. Liver NFAT5, BGT-1, SMIT, and aldose reductase total protein levels were visualized at three sampling points: control, 24 h anoxia, and 4 h recovery. See ‘Materials and Methods’ for more extensive definitions of the sampling points. Westerns blots and Coomassie total protein loading controls representative of the results are shown for the three sampling points. Also shown are histograms with mean standardized band densities (±S.E.M., *n* = 4 independent protein isolations from different animals). Data was analyzed using a one-way analysis of variance with a post hoc Tukey’s test (*p* < 0.05); for each parameter measured, values that are not statistically different from each other share the same letter notation. NFAT5, Nuclear Factor of Activated T Cells 5; BGT-1, betaine transporter 1; SMIT, sodium/myo-inositol cotransporter.

## Results

### Analysis of protein levels during anoxia-recovery

**Figure 3:**
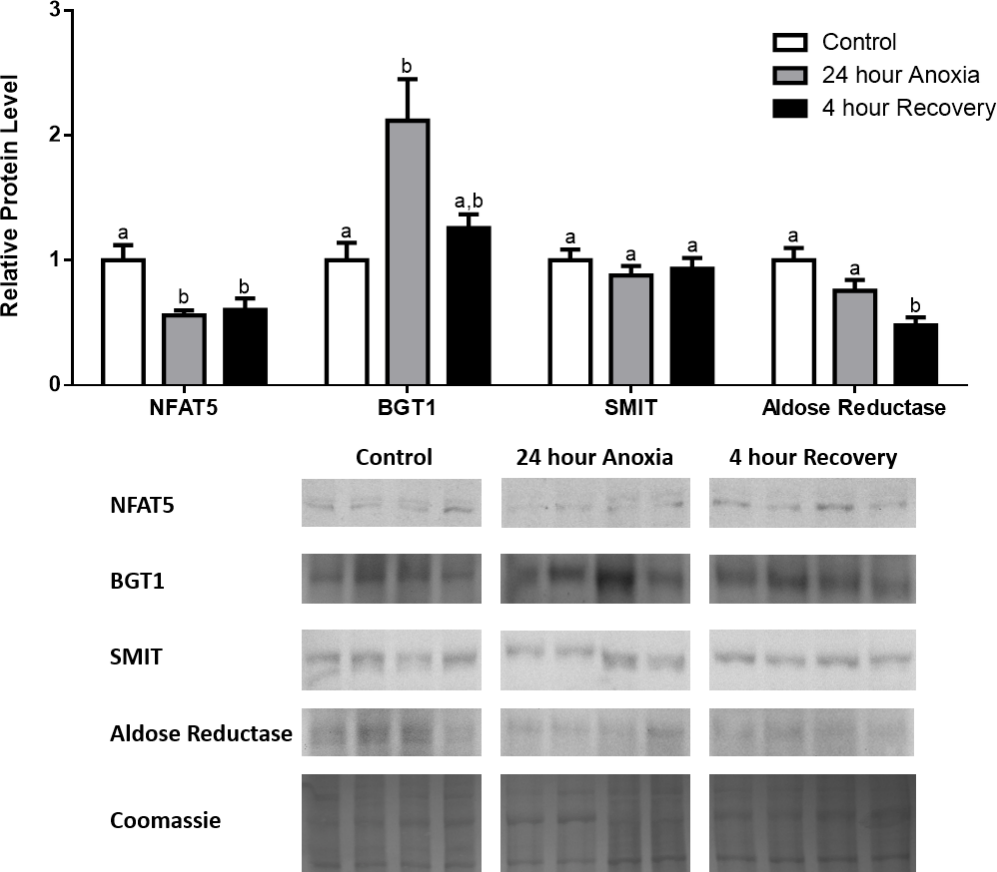
Changes in NFAT5, BGT-1, SMIT, and aldose reductase total protein levels in kidney during anoxia-recovery stress in *R. sylvatica*. Kidney NFAT5, BGT-1, SMIT, and aldose reductase total protein levels were visualized at three sampling points: control, 24 h anoxia, and 4 h recovery. See ‘Materials and Methods’ for more extensive definitions of the sampling points. Westerns blots and Coomassie total protein loading controls representative of the results are shown for the three sampling points. Also shown are histograms with mean standardized band densities (±S.E.M., *n* = 4 independent protein isolations from different animals). Data was analyzed using a one-way analysis of variance with a post hoc Tukey’s test (*p* < 0.05); for each parameter measured, values that are not statistically different from each other share the same letter notation. NFAT5, Nuclear Factor of Activated T Cells 5; BGT-1, betaine transporter 1; SMIT, sodium/myo-inositol cotransporter.

In the anoxic muscle, NFAT5 and BGT-1 levels increased relative to control by 0.7- and 0.9-fold relative to control, respectively. Aldose reductase levels decreased by 37% relative to control. SMIT levels in anoxic muscle did not change significantly from control levels. Following recovery from anoxia, muscle NFAT and BGT-1 levels decreased significantly from anoxia. SMIT levels increased by 1.04-fold relative to control, and these levels are significantly increased relative to anoxic muscle as well. Aldose reductase levels also continued to decrease relative to control by 48%, although this decrease was not significant relative to anoxia levels (Fig. 1). In the anoxic liver, NFAT5 levels decreased relative to control by 41%. BGT-1, SMIT, and aldose reductase levels did not change significantly from control levels. Following recovery from anoxia, liver NFAT5 levels were significantly decreased relative to control by 34%. After recovery, aldose reductase levels in liver decreased from control (by 43%) and from anoxia. BGT-1 and SMIT levels on the other hand did not change significantly in recovery liver relative to control or anoxic liver (Fig. 2). In the anoxic kidney, NFAT5 levels decreased by 44% relative to control. In contrast, BGT-1 levels increased by 1.1-fold relative to control. SMIT and aldose reductase levels did not change significantly relative to control. In kidney recovered from anoxia, NFAT5 levels did not change from anoxic levels, but they were still decreased from control levels by 40%. Similarly, aldose reductase levels were also decreased in kidney recovered from anoxia by 52% relative to control, and this decrease was also significant relative to anoxia. On the other hand, BGT-1 and SMIT levels did not change significantly in kidney recovered from anoxia relative to control and anoxic kidney (Fig. 3).

### Analysis of protein levels during dehydration–rehydration

**Figure 4:**
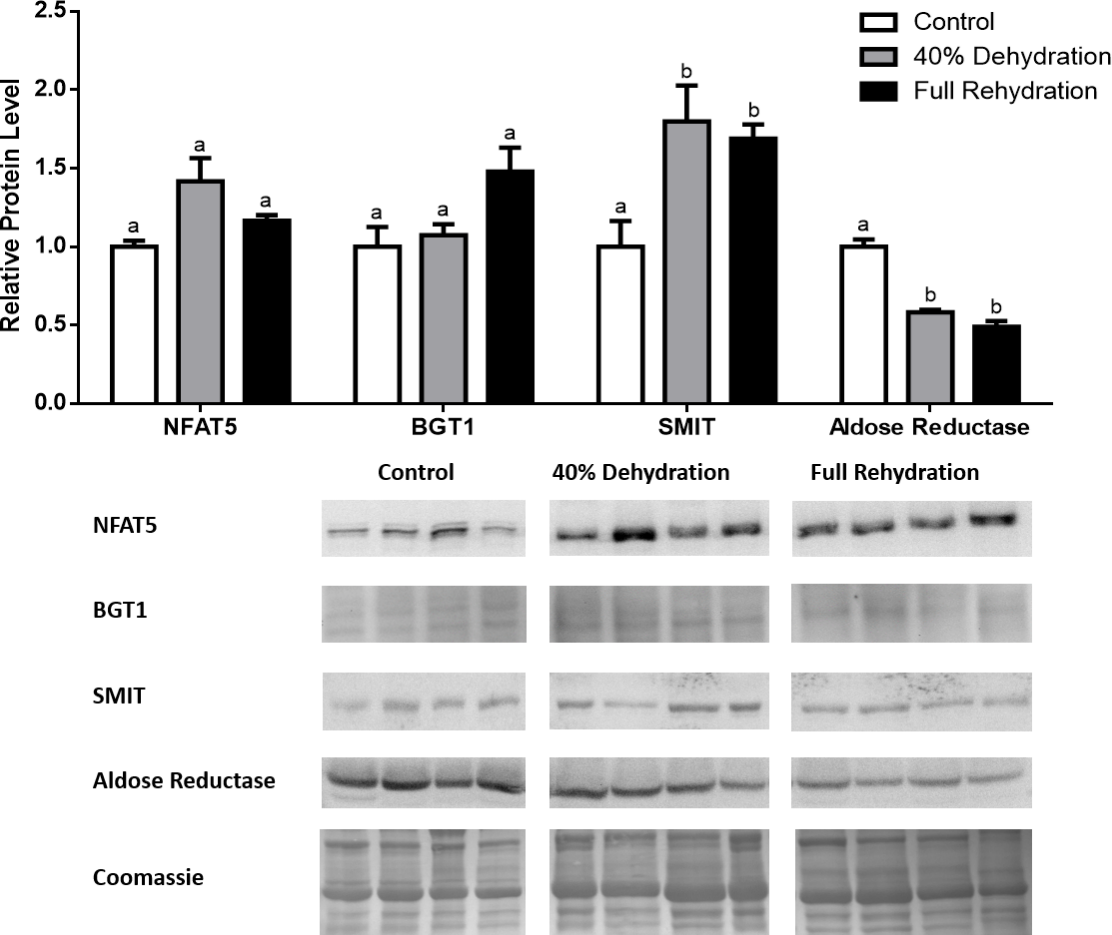
Changes in NFAT5, BGT-1, SMIT, and aldose reductase total protein levels in muscle during dehydration–rehydration stress in *R. sylvatica*. Muscle NFAT5, BGT-1, SMIT, and aldose reductase total protein levels were visualized at three sampling points: control, 40% dehydration, and full rehydration. See ‘Materials and Methods’ for more extensive definitions of the sampling points. Westerns blots and Coomassie total protein loading controls representative of the results are shown for the three sampling points. Also shown are histograms with mean standardized band densities (±S.E.M., *n* = 4 independent protein isolations from different animals). Data was analyzed using a one-way analysis of variance with a post hoc Tukey’s test (*p* < 0.05); for each parameter measured, values that are not statistically different from each other share the same letter notation. NFAT5, Nuclear Factor of Activated T Cells 5; BGT-1, betaine transporter 1; SMIT, sodium/myo-inositol cotransporter.

**Figure 5:**
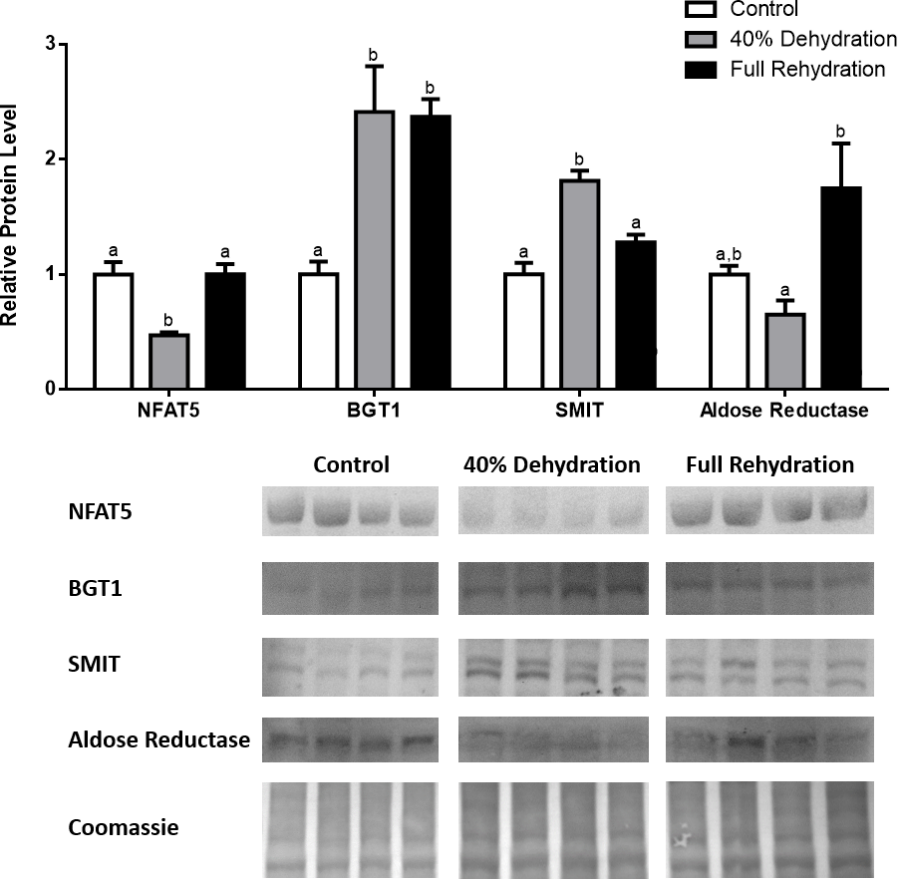
Changes in NFAT5, BGT-1, SMIT, and aldose reductase total protein levels in liver during dehydration–rehydration stress in *R. sylvatica*. Liver NFAT5, BGT-1, SMIT, and aldose reductase total protein levels were visualized at three sampling points: control, 40% dehydration, and full rehydration. See ‘Materials and Methods’ for more extensive definitions of the sampling points. Westerns blots and Coomassie total protein loading controls representative of the results are shown for the three sampling points. Also shown are histograms with mean standardized band densities (±S.E.M., *n* = 4 independent protein isolations from different animals). Data was analyzed using a one-way analysis of variance with a post hoc Tukey’s test (*p* < 0.05); for each parameter measured, values that are not statistically different from each other share the same letter notation. NFAT5, Nuclear Factor of Activated T Cells 5; BGT-1, betaine transporter 1; SMIT, sodium/myo-inositol cotransporter.

**Figure 6:**
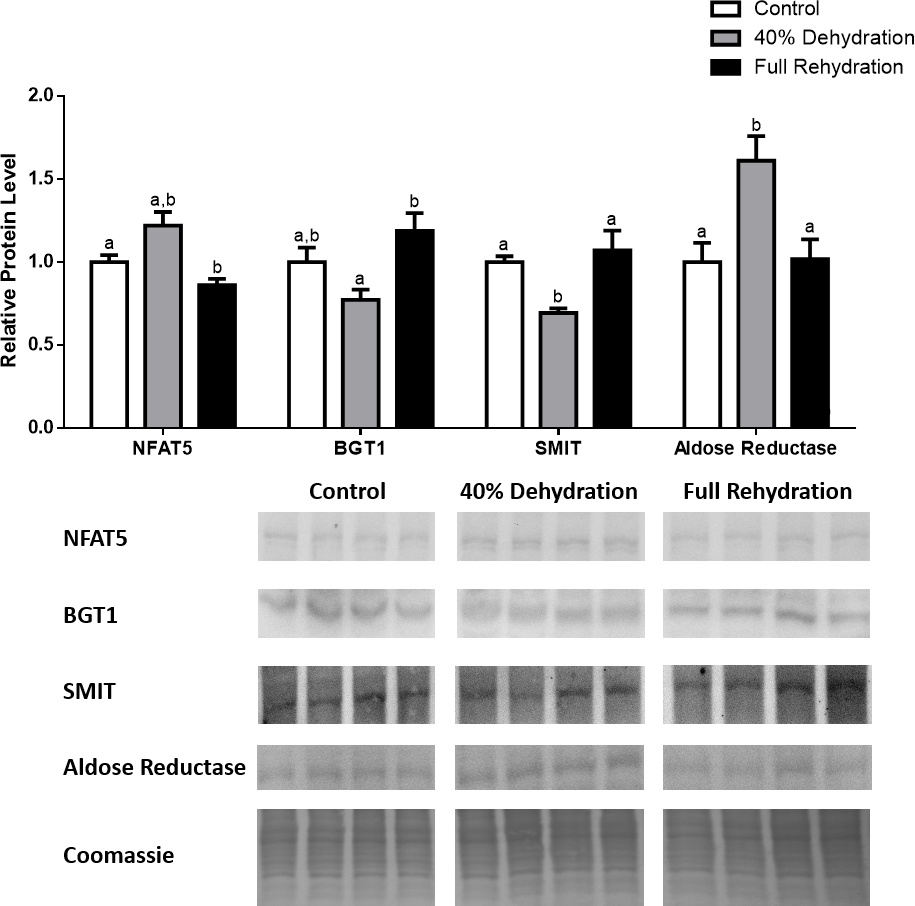
Changes in NFAT5, BGT-1, SMIT, and aldose reductase total protein levels in kidney during dehydration–rehydration stress in *R. sylvatica*. Kidney NFAT5, BGT-1, SMIT, and aldose reductase total protein levels were visualized at three sampling points: control, 40% dehydration, and full rehydration. See ‘Materials and Methods’ for more extensive definitions of the sampling points. Westerns blots and Coomassie total protein loading controls representative of the results are shown for the three sampling points. Also shown are histograms with mean standardized band densities (±S.E.M., *n* = 4 independent protein isolations from different animals). Data was analyzed using a one-way analysis of variance with a post hoc Tukey’s test (*p* < 0.05); for each parameter measured, values that are not statistically different from each other share the same letter notation. NFAT5, Nuclear Factor of Activated T Cells 5; BGT-1, betaine transporter 1; SMIT, sodium/myo-inositol cotransporter.

In dehydrated muscle, SMIT levels increased significantly by 0.8-fold relative to control. Aldose reductase levels on the other hand decreased by 42% relative to control. During rehydration, muscle SMIT levels remained elevated relative to control (0.69-fold), but these levels did not change from dehydrated muscle levels. Aldose reductase levels during rehydration remained significantly decreased relative to control (51%), although rehydration levels were not significantly different from dehydration. In *R. sylvatica* muscle, NFAT5 and BGT-1 levels were not different between control, dehydration, and rehydration (Fig. 4). In dehydrated liver, NFAT5 levels decreased by 53% relative to control. In contrast, BGT-1 and SMIT levels increased relative to control by 1.4-fold and 0.8-fold, respectively. Aldose reductase levels did not change significantly relative to control levels. In rehydrated liver, NFAT5 levels returned to control levels and were significantly elevated over dehydrated liver levels. Liver BGT-1 levels remained elevated during rehydration, and were increased by 1.4-fold relative to control. However, these levels were not significantly different from dehydration. SMIT levels decreased from rehydration by 29% relative to dehydration, but rehydrated levels were not significantly different from control. Aldose reductase levels during rehydration were significantly elevated over dehydrated liver levels by 1.7-fold, but this increase was not significant relative to control (Fig. 5). In dehydrated kidney, SMIT levels are significantly decreased relative to control by 41%. Aldose reductase levels on the other hand increased by 0.6-fold relative to control. NFAT5 and BGT-1 levels did not change relative to control. In the rehydrated kidney, NFAT5 and aldose reductase levels were both decreased relative to dehydrated levels by 29% and 37%, respectively. However, the levels of both proteins did not differ relative to control. BGT-1 and SMIT levels on the other hand, increased at rehydration relative to dehydrated kidney levels, both by 0.5-fold. However, neither of these proteins changed relative to control as well (Fig. 6).

## Discussion

The present study furthers our understanding of the osmoregulatory mechanisms that can assist *R. sylvatica* in resisting cellular damage caused by dehydration and anoxia. Previous studies have identified that the production and transport of organic osmolytes such as betaine, sorbitol, and myo-inositol into cells help to maintain osmolarity and cell volume in a hypertonic environment (Yancey et al., 1982; Garcia-Perez & Burg, 1991). Aldose reductase is the enzyme that synthesizes sorbitol inside the cell. In addition, SMIT and BGT-1 transport myo-inositol and betaine, respectively, into the cell (Burg, Kwon & Kültz, 1997; Gallazzini et al., 2006). Aldose reductase, SMIT, and BGT-1 are all regulated specifically by NFAT5 in response to cell shrinkage (Takenaka et al., 1994; Ferraris et al., 1996; Ko et al., 1997; Lopez-Rodríguez et al., 1999).

Anoxia is a stress that is not often studied in the context of osmoregulation. During hypoxia however, NFAT5 has been shown to be upregulated in the kidney and placenta (Villanueva et al., 2012; Dobierzewska et al., 2015). One proposed mechanism of NFAT5-mediated osmoregulation during hypoxia is that NFAT5 has a protective role against hypoxia by improving tolerance for hypertonicity (Villanueva et al., 2012). Our results identified that in the anoxic muscle, NFAT5 and BGT-1 increased by 0.7- and 0.9-fold, respectively (Fig. 1). This suggests that NFAT5 may play a protective role against anoxia in the muscle through the regulation of betaine transport into the cell. SMIT on the other hand, increased during recovery from anoxia. This delayed response in SMIT upregulation may come as a result of post-transcriptional modifications, such as inhibition by microRNAs (miR). In fact, several miRs have been shown to be differentially regulated during freezing in *R. sylvatica* (Bansal, Luu & Storey, 2016). Aldose reductase displayed a different pattern of protein expression in comparison with the other targets, where its protein levels were significantly decreased by 37% and 41% during anoxia and recovery, respectively (Fig. 1). This interesting trend may be influenced by cofactor availability, as anoxia results in insufficient oxygen supply to power aerobic ATP and NADPH generation by the mitochondria (Hanukoglu & Rapoport, 1995; Hochachka et al., 1996; Catling et al., 2005). Therefore, there may be a downregulation of aldose reductase, as a result of decreased NADPH, and this may partially explain why there is a 20-fold increase in cellular glucose (the substrate of aldose reductase) in the frozen wood frog (Storey & Storey, 1984).

In the liver, we observed decreased expression of NFAT5 during anoxia and recovery, with decreased levels of aldose reductase during recovery from anoxia (Fig. 2). A reason why we did not observe decreased levels of aldose reductase during anoxia despite the decrease in NFAT5 levels could have been due to a delayed response in the liver, resulting from the need to metabolize excess glucose in the liver that could not be distributed in the bloodstream due to anoxia and the inhibition of blood flow (Storey & Storey, 2004b). In the kidney, NFAT5 and aldose reductase protein levels were both decreased during anoxia and recovery. However, BGT-1 levels surprisingly increased during anoxia by 0.9-fold (Fig. 3). The decreases in NFAT5 and aldose reductase during anoxia supports the hypothesis that due to inadequate oxidative ATP production, there is inadequate ion pumping by ATP-dependent proteins like the NA^+^-K^+^-ATPase (Garcia-Dorado et al., 2012; Khanna et al., 2014). This results in cellular swelling and possibly edema due to intracellular water influx (Yang, Davis & Chow, 2001). During freezing, we know that anoxia occurs as a result of ice formation and the cessation of heart rate, but the anoxic stress on its own may initiate an osmoregulatory response to combat cell swelling instead of shrinking (Holden & Storey, 1997; Storey & Storey, 2004b). A decrease in NFAT5-mediated aldose reductase expression along with a release of sorbitol may contribute to the defense against cellular swelling and bursting during anoxia. In fact, decreases in aldose reductase levels during anoxia or delayed decreases during recovery were seen in all three tissues exposed to anoxia-recovery conditions, thus implying that anoxia alone may promote cellular swelling, especially in the muscle and kidney, and decreased sorbitol production may be a defense mechanism (Figs. 1– 3). Although the levels of BGT-1 and SMIT increase during anoxia variably in the muscle and kidney, there may be a significant shortage of betaine and myo-inositol that are available to be transported into the cells due to low blood flow, which may also be a defense against severe cell swelling and bursting during anoxia. Furthermore, an explanation for the dramatic rise in BGT-1 levels in anoxic kidney despite a decrease in NFAT5 levels could be that there are other mechanisms of regulating translation and protein synthesis in the wood frog while its metabolic rate is depressed. One such mechanism is the storage of mRNAs in stress granules that could serve to store essential mRNAs for genes like BGT-1 so that translation of the transcripts can be rapidly initiated when BGT-1 is needed to transport what little betaine is available in the anoxic kidney to restore the osmotic gradient of the cell (Wilczynska et al., 2005).

The four targets quantified in this study have been studied most extensively in the context of dehydration, which is severe enough in the wood frog to cause cell shrinkage but not cell death, and it is believed that this protective mechanism is mediated by the NFAT5-dependent osmoregulatory mechanism (Churchill & Storey, 1994; Burg, Kwon & Kültz, 1997; Storey & Storey, 2004b; Gallazzini et al., 2006; Chen et al., 2009). The results from this study show that in the kidney, NFAT5 is modestly upregulated during 40% dehydration, and aldose reductase increased by 0.6-fold during dehydration as a result (Fig. 6). However, BGT-1 and SMIT levels did not increase during dehydration, and this could be due to the mRNA processing mechanisms described above, in addition to decreased blood flow and therefore substrate availability during dehydration. These results suggest that the wood frog may prioritize the production of sorbitol as the primary mechanism of osmoregulation in the kidney during dehydration. In the skeletal muscle, we also see a modest increase in NFAT5 protein levels during dehydration, and SMIT levels increase as a result (Fig. 4). However, similar to anoxic muscle, aldose reductase levels decreased during dehydration as well. This could be due to the mechanism described previously, whereby decreased blood flow to muscle due to dehydration leads to decreased oxygen delivery and production of NADPH, which is needed for aldose reductase to produce sorbitol from glucose. Hence, there is an accumulation of glucose is the frozen wood frog (Storey & Storey, 1984). In the liver, NFAT5 protein levels were surprisingly decreased during dehydration, but BGT-1 and SMIT levels both showed large increases (Fig. 5). Furthermore, aldose reductase levels increased during rehydration, possibly as a result of restored blood volume and flow to the liver, as well as a need to metabolize excess glucose in the liver. The fact that we see such discrepancies between NFAT5 levels and the protein levels of BGT-1 and SMIT stress the possibility that mRNA processing through miRs and stress granules play a vital role in regulating protein translation and mRNA stability in a tissue- and stress-dependent manner, and these mechanisms are only just being discovered in frogs (Wilczynska et al., 2005; Bansal, Luu & Storey, 2016).

Our findings have shown that osmoregulation through the NFAT5-mediated pathway is not only tissue specific, as we predicted, but it is specific to the stress being induced on that tissue as well. A study conducted by Zhang et al. (2003) supports our findings by showing that the mRNA expression of NFAT5, aldose reductase, BGT-1, and SMIT1 is specific to the tissue and condition being evaluated (Zhang et al., 2003). For example, they showed that when rats were exposed to a hypotonic solution (similar to that of our rehydration condition and possibly anoxia), NFAT5 and BGT-1 mRNA decrease in liver, and SMIT decreases in both liver and muscle. These results are similar to our protein expression data, with the exception of NFAT5, possibly due to either mRNA processing, regulation of translation, or the unique ability of wood frogs to adapt to stressful conditions in comparison with mammals with less sophisticated osmoregulatory capabilities, such as rats. In *R. sylvatica*, there was a coordinated stress response of osmoregulation through the regulation of aldose reductase, BGT-1, and SMIT by NFAT5 in a tissue- and stress-dependent manner.

In conclusion, in wood frogs, the interplay between NFAT5 and aldose reductase seems to be especially important for osmoregulation as substrate availability is often limited during anoxia and dehydration, thus limiting the ability of BGT-1 and SMIT to perform their functions as transporters. These novel findings on the natural physiological stresses confronting the wood frog advance our knowledge of osmoregulatory mechanisms, and highlight the possibility of using this model to further study the molecular mechanisms underlying various physiological adaptations.

##  Supplemental Information

10.7717/peerj.2797/supp-1 Supplemental Information 1 Supplementary Table 1 - Raw data valuation analyzed from western blotting results Mean band densitometries (±S.E.M., *n* = 4 independent protein isolations from different animals) were calculated from representative bands that were standardized to the Coomassie total protein loading control lanes for each blot. The Data was then normalized to the mean of the control values, and these normalized values are shown above for each of the 4 targets at each of the 3 condtions in each of the 3 tissues, and were plotted and shown on histograms for Figs. 1–6 1-6. This data consists of either an *n* = 4 or *n* = 3 for each time point for each target because occasionally a sample needed to be removed from analysis because the protein was degraded or it was a clear outlier. Data (*n* = 3 or *n* = 4) were analyzed using a one-way analysis of variance with a post hoc Tukey’s test (*p* < 0.05); for each parameter measured, values that are not statistically different from each other share the same letter notation on the Figs. 1–6.

## Additional Information and Declarations

### Field Study Permissions

The following information was supplied relating to field study approvals (i.e., approving body and any reference numbers): All animal care, experimentation and euthanasia procedures are approved by the Carleton University Animal Care Committee (animal protocol no. 13683) in accordance to the guidelines set forth by the Canadian Council on Animal Care. The field permit is included within this animal protocol.

### Data Availability

The following information was supplied regarding data availability: The raw data has been supplied as a Supplementary File. The present study did indeed generate raw data upon which the statistical analysis and data interpretation were conducted. We have included all the raw data in the original manuscript (Table S1) so that readers will be able to review and reproduce the results.
